# A Novel Statistical Prognostic Score Model That Includes Serum CXCL5 Levels and Clinical Classification Predicts Risk of Disease Progression and Survival of Nasopharyngeal Carcinoma Patients

**DOI:** 10.1371/journal.pone.0057830

**Published:** 2013-02-28

**Authors:** Haibo Zhang, Weixiong Xia, Xing Lu, Rui Sun, Lin Wang, Lisheng Zheng, Yanfang Ye, Yingna Bao, Yanqun Xiang, Xiang Guo

**Affiliations:** 1 Department of Nasopharyngeal Carcinoma, Sun Yat-Sen University Cancer Center, Guangzhou, China; 2 State Key Laboratory of Oncology in Southern China, Guangzhou, China; Kinghorn Cancer Centre, Garvan Institute of Medical Research, Australia

## Abstract

**Background:**

Aberrant expression of C-X-C motif chemokine 5 (CXCL5) contributes to the progression of various cancers. This study analyzed the clinical significance of serum CXCL5 (sCXCL5) levels of nasopharyngeal carcinoma (NPC) patients, with the goal of building a novel prognostic score model.

**Experimental Design:**

Serum samples were collected prior to treatment from 290 NPC patients for the detection of sCXCL5 with ELISA. Half of the patients (n = 145) were randomly assigned to the training set to generate the sCXCL5 cutoff point using receiver operator characteristic (ROC) analysis, while the other half (n = 145) were assigned to the testing set for validation. Associations between sCXCL5 levels and clinical characteristics were analyzed. A prognostic score model was built using independent predictors derived from multivariate analysis. A concordance index (C-Index) was used to evaluate prognostic ability.

**Results:**

The sCXCL5 cutoff point was 0.805 ng/ml. Sex, age, histology, T classification, clinical classification and local recurrence were not associated with sCXCL5 levels. However, sCXCL5 levels were positively associated with N classification, distant metastasis and disease progression (*P*<0.05). A high sCXCL5 level predicted poor 6-year overall survival (OS), poor 6-year distant metastasis-free survival (DMFS), and poor 6-year progression-free survival (PFS). A prognostic score model was subsequently constructed based on sCXCL5 levels and clinical classification (C-C model), which are independent predictors of OS, DMFS, and PFS, as confirmed by the multivariate analysis. Furthermore, this novel model successfully divided the patients into four risk subgroups in the training set, the testing set and the entire set of patients. The C-Indices were 0.751 and 0.762 for the training set and the testing set, respectively.

**Conclusions:**

sCXCL5 level was determined to be an independent prognostic factor for NPC patients. The novel statistical C-C model, which includes sCXCL5 levels and clinical classification, could be helpful in predicting the prognosis of NPC patients.

## Introduction

Nasopharyngeal carcinoma (NPC) is the most common cancer originating from the nasopharynx. NPC differs significantly from other head and neck cancers in terms of its high rate of distant metastasis, undifferentiated histology, radiosensitivity and chemosensitivity. The highest incidence of NPC is found in Southeast Asia, especially in the Guangdong Province of China [1]. One of the significant risk factors for NPC is Epstein-Barr virus infection. This infection initiates a multi-step process that eventually progresses to the development of NPC [2], [3].

Enlarged cervical lymph nodes are the initial presentation in many NPC patients. Thus, NPC is usually diagnosed as a lymph node-metastatic disease. While NPC is relatively radiosensitive and chemosensitive, local-regional failure and distant metastasis are still the leading causes of treatment failure in this disease. With the development of irradiation techniques and chemoradiotherapy, the local-regional control rate for NPC has improved greatly in the past few decades, but the incidence of distant metastases has not decreased significantly [4], [5], reaching rates as high as 19% to 25% [6]–[10]. A clinical classification system is commonly used in clinical practice to predict the outcome of NPC cases. In addition to the clinical classification system, an increasing number of biomarkers have been used to more precisely assess the prognoses of NPC patients. However, patients with the same clinical classification might have different prognoses after receiving a similar treatment. Therefore, there is a growing need for a novel prognostic model that utilizes both biomarkers and clinical classification to identify those patients with a poor prognosis before treatment and permit the use of much more aggressive treatment to improve overall survival.

Inflammation plays an important role in cancer development and progression. C-X-C motif chemokine 5 (CXCL5; formerly epithelial neutrophil-activating peptide-78 or ENA-78) is a member of the CXC chemokine family. CXCL5 induces the chemotaxis of neutrophils, promotes angiogenesis, and is involved in the remodeling of connective tissue [11], [12]. Recently, CXCL5 was shown to promote the proliferation, migration, and invasion of various tumor cells *in vitro* and *in vi*vo [13]–[16]. For example, CXCL5 is overexpressed in oral squamous cell carcinoma, colorectal cancer, hepatocellular cancer, prostate cancer, gastric cancer and pancreatic cancer [14]–[20], with overexpression being associated with poor patient survival [15]–[17]. However, the association between serum CXCL5 (sCXCL5) expression and the prognosis of NPC patients is unclear.

In this study, we determined the sCXCL5 concentrations of 290 non-metastatic NPC patients before treatment and analyzed the associations between sCXCL5 concentrations and clinicopathological characteristics and prognosis. Based on these data, we constructed a novel prognostic score model based on clinical classification and sCXCL5 levels to predict the prognosis of NPC patients.

## Materials and Methods

### Patient Selection and Serum Collection

The Clinical Ethics Review Board of Sun Yat-Sen University Cancer Center approved this study. All of the patients signed informed consent documents prior to participating in this study.

Two hundred and ninety consecutive NPC patients who were newly diagnosed between July 2003 and August 2005 were recruited from Sun Yat-Sen University Cancer Center for this study. The eligibility criteria for inclusion in the study were as follows: an age of 18–65 years, pathological confirmation of undifferentiated non-keratinized or differentiated non-keratinized carcinoma of the nasopharynx, a Union for International Cancer Control (UICC) staging system 2002 clinical classification of I to IVb. The exclusion criteria included a history of anticancer therapy, pregnancy or lactation, and the presence of contraindications for receiving chemotherapy or radiotherapy.

Blood samples were obtained by venipuncture prior to anticancer therapy, centrifuged at 3000 rpm for 10 min and then frozen at –80°C until analysis.

### Pretreatment Evaluation of the Patients

All of the patients underwent a pretreatment evaluation that included a precise clinical examination of the head and neck region, fiber optic nasopharyngoscopy, head and neck MRI, chest X-ray, ultrasonography of the abdominal region, bone scan, and a complete blood count and biochemical profile.

### Patient Treatment

All of the patients received continuous definitive radiotherapy consisting of 2 Gy/fraction/day, five days/week. This therapy was delivered by a linear accelerator (6–8 MV) for 6–8 weeks. The range of radiation doses delivered to the primary tumor site was 60–78 Gy, while the doses delivered to the lymph node-positive regions and the lymph node-negative areas ranged from 60–70 Gy and 50–60 Gy, respectively.

In addition to radiotherapy, 236 patients with class III, IVa, or IVb disease received platinum-based chemotherapy. Of this group, 90 patients received inductive chemotherapy and radiotherapy, 55 patients received concurrent chemoradiotherapy, and 91 patients received inductive chemotherapy and concurrent chemoradiotherapy. Inductive chemotherapy consisted of 2 cycles of 5-ﬂuorouracil (4 g/m^2^) and cisplatin (80 mg/m^2^) every 3 weeks. Concurrent chemoradiotherapy consisted of 2 to 3 cycles of high-dose cisplatin (80 mg/m^2^) for 3 weeks. Four of the patients with class III disease refused chemotherapy and received radiotherapy only, and 1 patient with class II disease received concurrent chemoradiotherapy.

### Patient Follow-up

After the completion of therapy, all of the patients attended follow-up visits at 3-month intervals for the first 3 years, every 6 months for the fourth and fifth years, and annually thereafter. The primary end point of the study was overall survival (OS). The secondary end points were distant metastasis-free survival (DMFS), local–regional recurrence-free survival (LRRFS) and progression-free survival (PFS). These end points were defined as follows: OS, survival during the follow-up period; DMFS, survival without distant metastasis; LRRFS, survival without persistence or recurrence in the nasopharynx or cervical lymph nodes; and PFS, survival without local-regional failure or distant metastasis.

### ELISA Detection of sCXCL5 Levels

Serum CXCL5levels were measured with a commercially available ELISA kit (Quantikine Human ENA-78; R&D Systems, Minneapolis, MN, USA) in accordance with the manufacturer’s instructions. The measurements were performed in triplicate, and the data were summarized as the mean±SD.

### Statistical Analyses

The Statistical Package for Social Sciences, version 16.0 (SPSS, Chicago, IL, USA) was used for all of the statistical analyses and for the generation of a random number table for assigning patients to either the training or testing sets. A receiver operating characteristic (ROC) curve analysis was subjected to the selection of cutoff points of sCXCL5 concentration for OS. The chi-squared test was employed to compare data among groups. Cumulative survival rates were set with the life-table method. Differences in survival probabilities were determined by Kaplan-Meier analysis and the log-rank test. A multivariate analysis was performed with the Cox proportional hazards model (enter method) to analyze the factors related to prognosis. All of the statistical tests were two-sided, and *P*-values<0.05 were considered statistically significant. For the analyses involving multiple testing, adjusted *P*-values<0.017 were considered statistically significant.

One hundred and forty-five of the 290 patients were randomly assigned to the training set, which was used to generate the sCXCL5 cutoff point, evaluate the prognostic factors, and develop a prognostic score model. The remaining 145 patients were assigned to the testing set for data validation.

## Results

### Patient Clinical Characteristics

The latest patient follow-up visit occurred in May 2012. The time range for follow-up visits was between 8 and 105 months, with a median of 80 months. The follow-up rates at 1, 3, and 6 years were 100%, 100%, and 94.827%, respectively. One hundred and six patients died during the follow-up period, with 101 patients dying due to local-regional relapse and/or distant metastasis and 5 dying of non-neoplastic diseases. A total of 123 patients experienced disease recurrence: 73 developed distant metastasis, 40 developed local-regional relapse, and 10 developed both distant metastasis and local-regional relapse. The most frequent metastatic sites were the bone, liver, and lung, occurring in 29, 20, and 14 patients, respectively. Nineteen patients suffered from multiple organ metastases, and 1 patient had metastasis to the mediastinal and retroperitoneal lymph nodes. The clinicopathological characteristics of the 290 patients are summarized in Table 1.

**Table 1:** The clinicopathological characteristics of the NPC patients in the training and testing sets and their association with sCXCL5 levels.

	All patients	Training set (n = 145)			Testing set (n = 145)		
	n = 290 (%)	High (%)(n = 75)	Low (%)(n = 70)	*P*	High (%)(n = 90)	Low (%)(n = 55)	*P*
Age (years)							
<50	199 (69)	53 (71)	49 (70)	0.930	59 (65)	38 (69)	0.661
≥ 50	91 (31)	22 (29)	21 (30)		31 (35)	17 (31)	
Gender							
Male	204 (70)	53 (71)	50 (71)	0.919	61 (68)	40 (73)	0.529
Female	86 (30)	22 (29)	20 (29)		29 (32)	15 (27)	
Histological type							
D	36 (12)	9 (12)	7 (10)	0.190	13 (14)	7 (13)	0.771
U	254 (88)	66 (88)	63 (90)		77 (86)	48 (87)	
Tumor classification							
T1–2	79 (27)	2 4(32)	22 (31)	0.941	23 (26)	10 (18)	0.304
T3–4	211 (73)	51 (68)	48 (69)		67 (74)	45 (82)	
Nodal classification							
N0–1	156 (54)	35 (47)	45 (64)	0.033	41 (46)	35 (64)	0.034
N2–3	134 (46)	40 (53)	25 (36)		49 (54)	20 (36)	
Clinical classification							
I–II	50 (17)	12 (16)	16 (23)	0.296	14 (16)	8 (15)	0.869
III–IVb	240 (83)	63 (84)	54 (77)		76 (84)	47 (85)	
Chemotherapy							
No	53 (18)	12 (16)	15 (21)	0.527	16 (18)	10 (18)	0.292
Inductive	90 (31)	21 (28)	19 (27)		28 (31)	22 (40)	
Concurrent	56 (19)	13 (17)	16 (23)		21 (23)	6 (11)	
Inductive+ concurrent	91 (31)	29 (39)	20 (29)		25 (28)	17 (31)	
Local–regional recurrence							
No	240 (83)	58 (77)	55 (79)	0.857	76 (84)	51 (92)	0.142
Yes	50 (17)	17 (23)	15 (21)		14 (16)	4 (7)	
Distant metastasis							
No	207 (71)	47 (63)	57 (81)	0.012	58 (64)	45 (82)	0.025
Yes	83 (29)	28 (37)	13 (19)		32 (36)	10 (18)	
Progression							
No	167 (58)	32 (43)	44 (63)	0.015	49 (54)	42 (76)	0.008
Yes	123 (42)	43 (57)	26 (37)		41 (46)	13 (24)	

Abbreviations: NPC, nasopharyngeal carcinoma; sCXCL5, serum CXCL5; D, differentiated non-keratinized carcinoma; U, undifferentiated non-keratinized carcinoma.

### Relationship between sCXCL5 Levels and Clinical Characteristics of NPC Patients

The sCXCL5 concentrations in the 290 NPC patients ranged from 0.135 ng/ml to 3.058 ng/ml, with a mean of 0.980±0.48 ng/ml and a median of 0.828 ng/ml. We used the training set to construct ROC curves for death events and censors to identify the impact of sCXCL5 levels on the survival of NPC patients, selecting 0.805 ng/ml as the cutoff point for the subsequent binary variable analysis. Sex, age, histological type, T classification, clinical classification and local recurrence had no impact on sCXCL5 levels. However, the sCXCL5 level was positively associated with advanced N classification, distant metastasis and disease progression (*P*<0.05). A similar association was verified in the testing set (Table 1).

### Serum CXCL5 Levels in Predicting the Survival of NPC Patients

**Figure 1:**
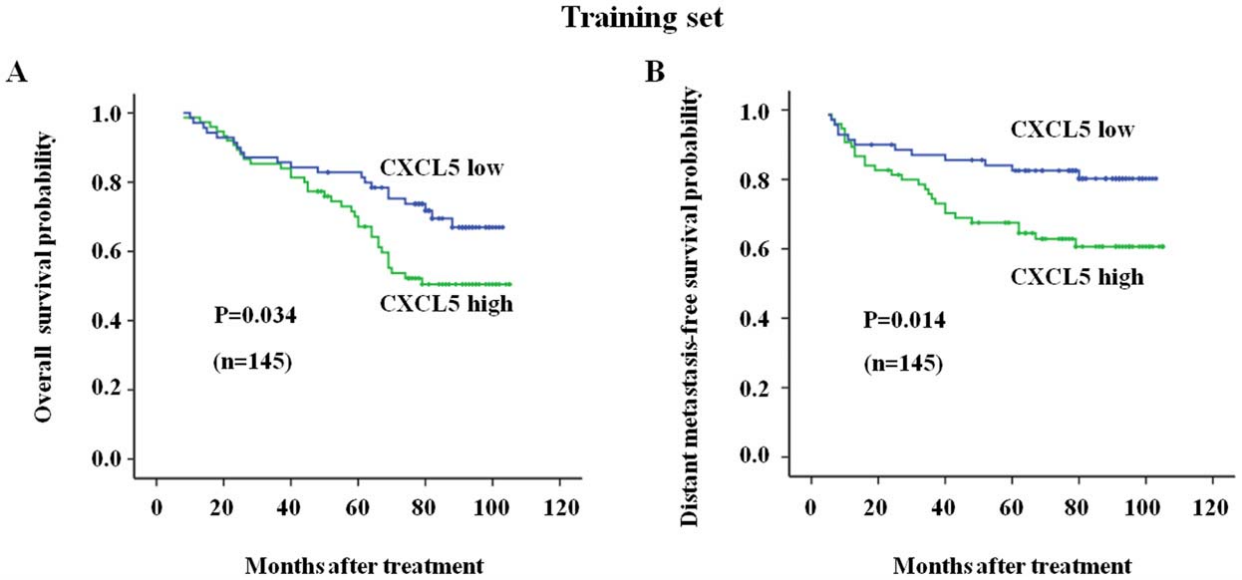
The survival curves for the nasopharyngeal carcinoma patients with high/low serum CXCL5-levels in the training set. A high sCXCL5 level correlated with poor overall survival and distant metastasis-free survival rates in the training set patients. (A) The overall survival rate was significantly higher in the low sCXCL5 level patients; (B) The distant metastasis-free survival rate was significantly higher in the low sCXCL5 level patients. Low sCXCL5 level, n = 70; high sCXCL5 level, n = 75.

In the training set, the 6-year-OS rates for the low sCXCL5 level group and the high sCXCL5 level group were 75% and 54%, respectively (*P* = 0.034). The 6-year-DMFS rates for the low sCXCL5 level group and the high sCXCL5 level group were 82% and 63% (*P* = 0.014), respectively (Figure 1 shows the complete follow-up). The 6-year-PFS rates for the low sCXCL5 level group and the high sCXCL5 level group were 65% and 45% (*P* = 0.021), respectively. However, there were no significant differences in the 6-year-LRRFS rates of the two groups, with 75% and 77% (*P* = 0.955) LRRFS rates for the high and low sCXCL5 level groups, respectively.

### Cox Proportional Hazards Model Analyses

Cox proportional hazards model was used for univariate analyses to determine if age, gender, histologic type, T classification, N classification, clinical classification, or sCXCL5 level was a prognostic factor for OS, DMFS or PFS in the training set. The results showed that T classification, N classification, clinical classification and sCXCL5 levels were significantly associated with OS, DMFS, and PFS in NPC patients (Table 2).

**Table 2:** Univariate analysis with the Cox proportional hazards model for the OS, DMFS, and PFS of the NPC patients in the training set (n = 145).

Prognosis	Wald	*P*	Exp(B)	95% CI for Exp(B)			
				Lower	Upper		
OS							
Age (years) ≥50 vs.<50	1.191	0.273	1.372	0.779		2.417	
Gender Female vs. Male	0.530	0.462	0.799	0.440		1.452	
Histological type U vs. D	0.152	0.701	0.852	0.376		1.931	
T classification T2-4 vs. T1	20.923	<0.001^△^	2.284	1.603		3.255	
N classification N1-3vs. N0	12.529	<0.001^△^	1.765	1.288		2.417	
Clinical classification II-IVb vs. I	25.912	<0.001^△^	3.249	2.067		5.106	
sCXCL5 level High vs. Low	5.893	0.015	2.013	1.148		3.529	
DMFS							
Age (years) ≥50 vs.<50	0.374	0.541	0.796	0.384		1.652	
Gender Female vs. Male	0.111	0.739	1.119	0.577		2.171	
Histological type U vs. D	0.111	0.739	0.848	0.320		2.243	
T classification T2-4 vs. T1	2.698	0.100^△^	1.346	0.944		1.920	
N classification N1-3 vs. N0	35.143	<0.001^△^	3.342	2.243		4.981	
Clinical classification II-IVb vs. I	7.949	0.005^△^	1.958	1.227		3.124	
sCXCL5 level High vs. Low	5.322	0.021	2.209	1.127		4.330	
PFS							
Age (years) ≥50 vs.<50	0.102	0.749	0.916	0.533		1.572	
Gender Female vs. Male	0.157	0.692	0.898	0.529		1.525	
Histological type U vs. D	0.359	0.549	0.800	0.385		1.661	
T classification T2-4 vs. T1	13.884	<0.001^△^	1.731	1.297		2.309	
N classification N1-3 vs. N0	21.655	<0.001^△^	1.988	1.488		2.656	
Clinical classification II-IVb vs. I	19.064	<0.001^△^	2.249	1.563		3.237	
sCXCL5 level High vs. Low	5.504	0.019	1.819	1.103		2.998	

△, adjusted P-values <0.017 were considered statistically significant. Abbreviations: OS, overall survival; DMFS, distant metastasis-free survival; PFS, progression-free survival; NPC, nasopharyngeal carcinoma; sCXCL5, serum CXCL5; D, differentiated non-keratinized carcinoma; U, undifferentiated non-keratinized carcinoma; CI, confidence interval.

Multivariate analyses using the Cox proportional hazards model was further conducted in the training set to determine the independent prognostic factors of NPC patients, including all of the factors analyzed in the univariate analysis. The results showed that T classification, N classification, and sCXCL5 levels were independent predictors of OS.

The overlap between clinical classification and T/N classification required the application of further Cox proportional hazards model analyses, which included clinical classification but not T classification or N classification, as well as the rest of the clinical characteristics, to the training set. The results showed that both clinical classification and sCXCL5 level were independent predictors of OS (*P*<0.05, Table 3).

**Table 3:** Multivariate analysis with the Cox proportional hazards model for the OS of the NPC patients in the training set (n = 145).

Analysis	Prognosis	Wald	*P*	Exp(B)	95% CI for Exp(B)	
					Lower	Upper
I	OS					
	Age (years) ≥50 vs.<50	2.780	0.095	1.620	0.919	2.857
	Gender Female vs. Male	0.095	0.759	0.911	0.503	1.649
	Histological type U vs. D	0.410	0.522	0.769	0.343	1.720
	T classification T2-4 vs. T1	6.939	0.008^△^	2.584	1.275	5.237
	N classification N1-3 vs. N0	11.823	0.001^△^	2.025	1.354	3.027
	Clinical classification II- IVb vs. I	0.163	0.686^△^	1.191	0.510	2.778
	sCXCL5 level High vs. Low	5.834	0.016	2.014	1.141	3.553
II	OS					
	Age (years) ≥50 vs.<50	1.200	0.446	1.010	0.984	1.038
	Gender Female vs. Male	0.541	0.470	0.802	0.441	1.459
	Histological type U vs. D	0.147	0.607	0.809	0.360	1.818
	Clinical classification II- IVb vs. I	26.083	<0.001^△^	3.217	2.045	5.062
	sCXCL5 level High vs. Low	5.970	0.014	2.008	1.145	3.523

△, adjusted P-values <0.017 were considered statistically significant. Abbreviations: OS, overall survival; NPC, nasopharyngeal carcinoma; sCXCL5, serum CXCL5; D, differentiated non-keratinized carcinoma; U, undifferentiated non-keratinized carcinoma; CI, confidence interval.

### The Prognostic Score Model

Based on the above analysis, we built a new prognostic score model that utilized both NPC clinical classification and sCXCL5 level (C-C model) to predict NPC prognosis in the training set. Based on the hazard ratio derived from the multivariate analysis for OS, a score of 1 to 3 was assigned to clinical classification and a score of 1 to 2 was assigned to sCXCL5 level. Because the survival rates for the class I and II NPC patients were not significantly different (100% for clinical class I vs. 92% for clinical class II, *P* = 0.648), a score of 1 was assigned to both clinical classification I and clinical classification II. The total scores for each patient were calculated by adding the two scores together; therefore, the total scores ranged from 2 to 5 (mean = 3.7 and median = 4). The patients were divided into 4 risk subgroups based on their total score: low risk (L, score 2, 11.0% of all 290 patients); intermediate-low risk (IL, score 3, 26.9% of all 290 patients); intermediate-high risk (IH, scores 4, 42.8% of all 290 patients) and high risk (H, score 5, 19.3% of all 290 patients).

**Figure 2:**
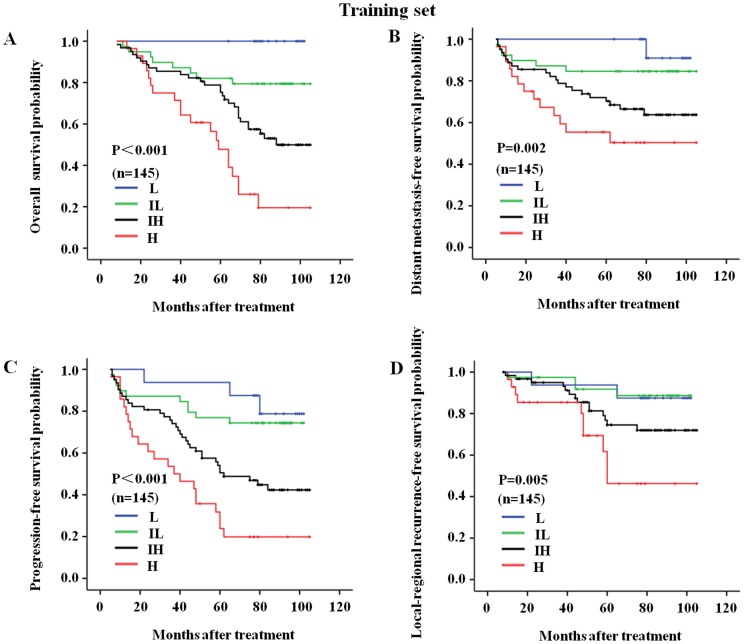
The C-C model-derived survival curves for the nasopharyngeal carcinoma patients in the training set. The follow-up prognoses of the nasopharyngeal carcinoma patients in the training set were clearly identified by the four risk subgroups of the C-C model. (A) The overall survival curves for the L, IL, IH, and H risk subgroups of the C-C model; (B) The distant metastasis-free survival curves for the L, IL, IH, and H risk subgroups of the C-C model; (C) The progression-free survival curves for the L, IL, IH, and H risk subgroups of the C-C model; and (D) The local-regional recurrence-free survival curves for the L, IL, IH, and H risk subgroups of the C-C model. L, low-risk, n = 16; IL, intermediate-low-risk, n = 39; IH, intermediate-high-risk, n = 62; H, high-risk, n = 28.

**Figure 3:**
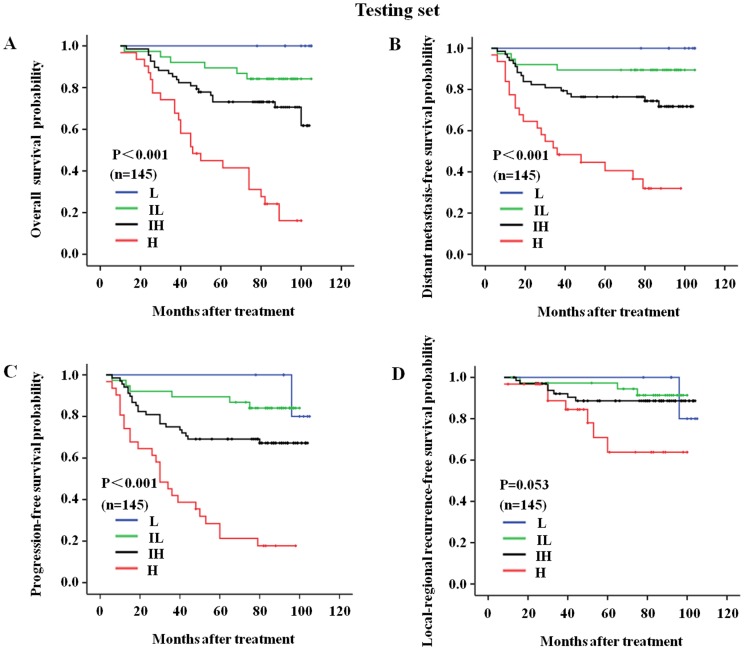
The C-C model-derived survival curves for the nasopharyngeal carcinoma patients in the testing set. The follow-up prognoses of the nasopharyngeal carcinoma patients in the testing set were clearly identified by the four risk subgroups of the C-C model. (A) The overall survival curves for the L, IL, IH, and H risk subgroups of the C-C model; (B) The distant metastasis-free survival curves for the L, IL, IH, and H risk subgroups of the C-C model; (C) The progression-free survival curves for the L, IL, IH, and H risk subgroups of the C-C model; and (D) The local-regional recurrence-free survival curves for the L, IL, IH, and H risk subgroups of the C-C model. L, low risk, n = 8; IL, intermediate-low risk, n = 38; IH, intermediate-high risk, n = 68; H, high risk, n = 31.

**Figure 4:**
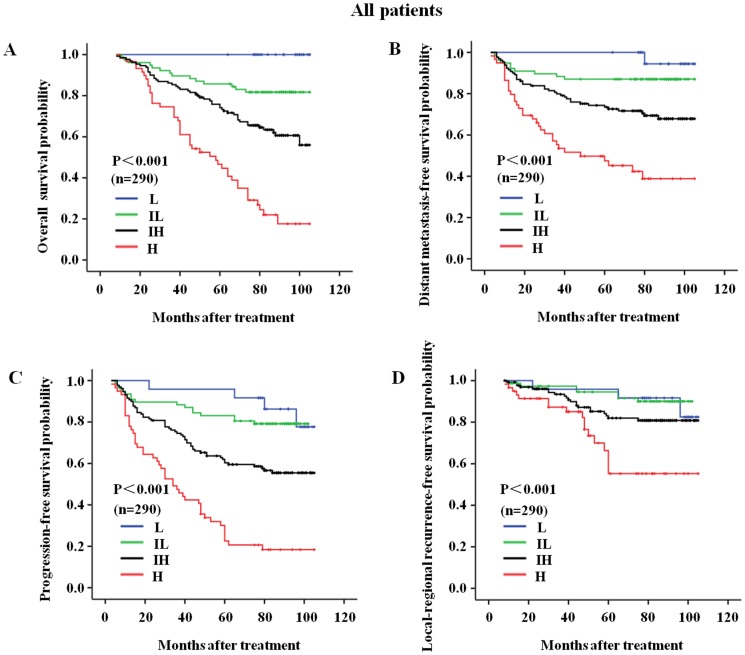
The C-C model-derived survival curves for all 290 nasopharyngeal carcinoma patients. The follow-up prognoses of all 290 nasopharyngeal carcinoma patients were clearly identified by the four risk subgroups of C-C model. (A) The overall survival curves for the L, IL, IH, and H risk subgroups of the C-C model; (B) The distant metastasis-free survival curves for the L, IL, IH, and H risk subgroups of the C-C model; (C) The progression-free survival curves for the L, IL, IH, and H risk subgroups of the C-C model; and (D) The local-regional recurrence-free survival curves for the L, IL, IH, and H risk subgroups of the C-C model. L, low risk, n = 24; IL, intermediate-low risk, n = 77; IH, intermediate-high risk, n = 130; H, high-risk, n = 59.

We further performed survival analyses for the patients in the training set, the testing set and all of the patients combined, with the results indicating that the OS, DMFS, LRRFS, and PFS curves discriminated between the four risk subgroups in the C-C model more clearly than clinical classification alone. In the training set, the 6-year-OS rates were 100%, 79%, 61%, and 27% for the L, IL, IH and H risk groups, respectively. These rates were 100%, 92%, 70%, and 41% for clinical class I, II, III and IVa-b patients, respectively (Table 4; Figures 2, 3, 4 show the complete follow-up). The C-Indices for clinical classification in the training set and testing set were 0.742 and 0.733, respectively, while these values were 0.751 and 0.762 for the C-C model. These results confirmed that the C-C model was more precise in predicting the prognosis of NPC patients than clinical classification alone.

**Table 4:** The C-C model-derived 6-year OS, DMFS, PFS, and LRRFS for the L, IL, IH, and H risk patient groups.

6-yearsurvival (%)	Training set (n = 145)					Testing set (n = 145)					All patients (n = 290)				
	L	IL	IH	H	*P*	L	IL	IH	H	*P*	L	IL	IH	H	*P*
OS	100	79	61	27	<0.001	100	87	73	42	<0.001	100	83	67	35	<0.001
DMFS	100	85	66	49	<0.001	100	89	76	40	<0.001	100	87	72	45	<0.001
PFS	88	74	49	20	<0.001	100	87	69	21	<0.001	92	80	59	21	<0.001
LRRFS	88	88	75	45	<0.001	100	94	89	64	<0.001	92	92	82	55	<0.001

Abbreviations: OS, overall survival; DMFS, distant metastasis-free survival; PFS, progression-free survival; LRRFS, local–regional recurrence-free survival; L, low risk group; IL, intermediate-low risk; IH, intermediate-high risk; H, high-risk.

## Discussion

In this study, we were the first to determine the sCXCL5 concentrations of NPC patients and analyze the associations between sCXCL5 levels and clinicopathological characteristics and prognosis. A high sCXCL5 level was associated with advanced lymph node classification, distant metastasis and tumor progression, findings that were verified in the testing set. We found that the OS and DMFS of NPC patients with high sCXCL5 levels were significantly poorer than the OS and DMFS of NPC patients with low sCXCL5 levels. The multivariate analysis further confirmed that clinical classification and sCXCL5 level were independent predictors of the OS, DMFS and PFS of NPC patients. We used clinical classification and sCXCL5 levels to build a novel statistical model (C-C model) to predict the prognosis of NPC patients. The C-C model classified NPC patients into 4 risk subgroups and was more effective in predicting the prognosis for NPC patients than clinical classification alone. This analysis also indicated that sCXCL5 level is an independent prognostic factor for NPC patients and that the C-C model could be applied in clinical practice to achieve a more accurate prognostic prediction than clinical classification alone. Thus, the use of this model in clinical practice would facilitate the individualized treatment of NPC patients in the future.

Various chemokines play important roles in the regulation of tumor progression. CXCL5 belongs to the glutamic acid-leucine-arginine (ELR) tripeptide motif cysteine-X-cysteine (CXC) chemokines. Depending on the presence or absence of the ELR motif at the NH_2_ terminus of the protein, CXC chemokines can be further classified as ELR-positive (ELR+) or ELR-negative (ELR−), respectively. The former can bind to CXC chemokine-receptor 2 (CXCR2). Because these molecules are mediators of angiogenesis [21], the expression of ELR+ and CXCR2 is associated with tumor progression [22]–[25]. Miyazaki et al. [14] found the transcriptional up-regulation of CXCL5 in the metastatic lymph nodes of head and neck squamous cell carcinoma patients. Park et al. [18] also reported that CXCL5 overexpression was associated with the lymph node metastasis of gastric cancer. Our current data similarly found an association between high sCXCL5 levels and advanced N classification. Studies have confirmed that high levels of CXCL5 are associated with high metastatic potential and poor survival rates in prostate cancer, colorectal cancer, hepatocellular carcinoma, and oral squamous cancers [13]–[16], [19]. In our study, we also found that NPC patients with high sCXCL5 levels developed more distant metastasis events and exhibited a more aggressive disease progression after treatment than patients with low sCXCL5 levels. This finding suggested that the pro-angiogenic property of CXCL5 might play an important role in the metastasis of NPC. To our knowledge, this study is the first report to evaluate the significance of sCXCL5 levels in the prognosis of NPC patients.

Several statistical prediction models have been proposed for metastatic NPC patients. For example, Tan et al. developed a new prognostic index score that utilized performance status, hemoglobin, disease-free interval months, and metastasis status at initial diagnosis and was useful in prognosticating and stratifying patients with disseminated NPC [26], [27]. Jin et al. [28] reported that a model built with hemoglobin, lactate dehydrogenase, alkaline phosphatase, Epstein–Barr virus DNA, and performance status could help guide the prognostication of metastatic NPC patients in epidemic areas. Cao et al. [29] found that a risk subset composed of viral capsid antigen-IgA titer, number of metastases, and secondary metastases may provide a more accurate and appropriate assessment of the prognosis for NPC patients with lung metastasis. However, clinical classification is widely used to predict the prognosis for non-metastatic NPC patients in clinical practice, despite the inaccuracy in predictions due to the heterogeneity of NPC. Statistical prediction models that included inflammatory biomarkers for non-metastatic NPC patients are rare. One such model used age, WHO histological type, serum lactate dehydrogenase, and tumor locations to predict local-regional control of non-disseminated NPC [30]. With the development of radiochemotherapy, the survival rate of NPC patients has improved significantly, but local-regional relapse and distant metastasis remain the major reasons for treatment failure in NPC patients. The ability to identify those patients with a very poor prognosis and high potential for metastasis before treatment is an urgent clinical problem.

In the current study, we built a novel C-C prognostic model from the patients in the training set by combining sCXCL5 level and clinical classification as parameters to predict the prognosis of NPC patients. The testing set was then used to verify the accuracy of this statistical model. We can better predict the disease progression and survival of NPC patients with the C-C model than with clinical classification alone. The C-Indices of clinical classification were lower than the C-Indices of the C-C model, as shown in the results. The survival curves for OS, DMFS, and PFS survival curves for the four risk subgroups of the C-C model were clearly distinguishable in the training set, the testing set, and a set containing all of the patients. With the exception of some overlap between the L and IL risk groups, the LRRFS can also be clearly discriminated with the C-C model. This finding strongly suggested that the new statistical model that used both sCXCL5 levels and the clinical classification system to accurately predict the prognosis of NPC patients was accurate and helpful in clinical practice.

However, we acknowledge that much more needs to be clarified before this model is eventually applied in clinical practice. For example, it will be interesting to investigate the correlation between sCXCL5 expression and functional changes in NPC tissue specimens, e.g., angiogenesis (blood vessel density) or Epstein-Barr virus infection status. We will incorporate these data into the statistical C-C model in future studies to better predict the risk of disease progression in NPC patients.
